# Impacting T-cell fitness in multiple myeloma: potential roles for selinexor and XPO1 inhibitors

**DOI:** 10.3389/fimmu.2023.1275329

**Published:** 2023-10-26

**Authors:** Adam F. Binder, Christopher J. Walker, Tomer M. Mark, Muhamed Baljevic

**Affiliations:** ^1^ Department of Medical Oncology, Division of Hematopoietic Stem Cell Transplant and Hematologic Malignancies, Thomas Jefferson University, Philadelphia, PA, United States; ^2^ Department of Translational Research, Karyopharm Therapeutics, Inc, Newton, MA, United States; ^3^ Division of Hematology and Oncology, Vanderbilt University Medical Center, Nashville, TN, United States

**Keywords:** multiple myeloma, SINE, T-cell exhaustion, CAR-T therapy, T-cell engagement, selinexor, XPO1

## Abstract

Competent T-cells with sufficient levels of fitness combat cancer formation and progression. In multiple myeloma (MM), T-cell exhaustion is caused by several factors including tumor burden, constant immune activation due to chronic disease, age, nutritional status, and certain MM treatments such as alkylating agents and proteasome inhibitors. Many currently used therapies, including bispecific T-cell engagers, anti-CD38 antibodies, proteasome inhibitors, and CART-cells, directly or indirectly depend on the anti-cancer activity of T-cells. Reduced T-cell fitness not only diminishes immune defenses, increasing patient susceptibility to opportunistic infections, but can impact effectiveness MM therapy effectiveness, bringing into focus sequencing strategies that could modulate T-cell fitness and potentially optimize overall benefit and clinical outcomes. Certain targeted agents used to treat MM, such as selective inhibitors of nuclear export (SINE) compounds, have the potential to mitigate T-cell exhaustion. Herein referred to as XPO1 inhibitors, SINE compounds inhibit the nuclear export protein exportin 1 (XPO1), which leads to nuclear retention and activation of tumor suppressor proteins and downregulation of oncoprotein expression. The XPO1 inhibitors selinexor and eltanexor reduced T-cell exhaustion in cell lines and animal models, suggesting their potential role in revitalizating these key effector cells. Additional clinical studies are needed to understand how T-cell fitness is impacted by diseases and therapeutic factors in MM, to potentially facilitate the optimal use of available treatments that depend on, and impact, T-cell function. This review summarizes the importance of T-cell fitness and the potential to optimize treatment using T-cell engaging therapies with a focus on XPO1 inhibitors.

## Introduction

Multiple myeloma (MM) is a malignancy of plasma cells and the second most frequent adult hematologic cancer in the US (1, 2). MM remains incurable despite the evolving therapeutic landscape of the last two decades, including the development of cornerstone agents such as proteasome inhibitors (PIs), immunomodulatory drugs (IMiDs), and monoclonal antibodies (mAbs). New agents with marked efficacy in treating relapsed and refractory MM include chimeric antigen receptor T-cells (CAR-T) and bispecific antibodies (3). Despite a high response rate to T-cell engaging therapies in MM, most patients experience relapse and require additional therapies before eventually succumbing to the disease. The success of T-cell engaging therapies may depend on the immune system status, specifically on the competence and fitness of T-cell populations, and the expression of different activating and inhibitory receptors (4).

CAR-T is designed to be a one-time therapy where a patient’s T-cells are collected and modified *ex vivo* into a functioning CAR-T product, then reintroduced into the patient. Crucial to developing a clinically effective biologic CAR-T product is a fit and numerically adequate T-cell population pre-leukapheresis. As such, consideration of drugs that do not negatively impact downstream T-cell health pre-leukapheresis may allow CAR-T function optimization (5). Likewise, mitigating T-cell exhaustion after the CAR-T product is infused into the patient to maintain the CAR-T effector function and achieve prolonged clinical potency remains a challenge, although maintenance therapies post-CAR-T therapy are being explored (6).

Studies have revealed that T-cell health is influenced by patient and disease characteristics and correlates with treatment outcomes (7). Alkylating agents and PIs, but not IMiDs, were most closely associated with inferior T-cell therapy clinical results (8). Though not yet studied in this context, selective inhibitors of nuclear export (SINE) compounds, referred to here as XPO1 inhibitors, may represent a class of T-cell sparing agents that could support later T-cell therapies. In addition to direct cytotoxicity against malignant cells, XPO1 inhibitors may modulate the immediate tumor immune microenvironment to promote T-cell fitness and reduce T-cell exhaustion, as demonstrated in preclinical melanoma and acute lymphoblastic leukemia (ALL) models (9, 10). Results of these studies indicate that dose and sequencing are critical to XPO1 inhibitor efficacy, as well as subsequent CAR-T therapy (9, 10); similar principles may apply to MM treatment.

This review highlights the importance of both T-cell fitness and optimizing treatment sequencing with therapies that affect T-cell engagement, fitness, and exhaustion (4). With this in mind, the evidence suggesting the ability of XPO1 inhibitors, such as selinexor, to enhance the effectiveness of T-cell engaging therapies is emphasized. These compounds, like IMiDs, present a small molecule option to enhance T-cell function dependent MM therapies (11).

## The importance of T-cell fitness

In the context of CAR-T therapy for hematological malignancies, T-cell fitness is the ability of a T-cell to generate an immune response that mediates the elimination of malignant cells and provides durable protection from relapse (5). T-cell exhaustion is defined by poor effector function, and characterized by reduced T-cell proliferation, increased expression of inhibitory receptors such as checkpoint inhibitors, and a distinct transcriptional signature (4, 12). In MM, disease progression is also associated with an altered T-cell repertoire including a reduction in bone marrow and peripheral blood effector T-cells, leading to an overall decrease in T-cell fitness (13). *In vitro* studies show that anti-BCMA CAR-T from healthy donors have superior long-term activity compared with those derived from RRMM patients who received CAR-T therapy (14).

T-cell exhaustion has been a crucial limiting factor in hematologic malignancy studies utilizing autologous CAR-T therapy (5, 15, 16). In a study of advanced, heavily pretreated patients with high-risk chronic lymphocytic leukemia (CLL) who received CD19 CAR-T therapy, biomarker profiles from autologous infusion products after CAR-T stimulation, demonstrated a correlation with the degree of treatment response (17). Higher expression of the T-cell exhaustion markers programmed cell death protein (PD)-1, T-cell immunoglobulin and mucin domain-containing protein 3 (TIM3), and lymphocyte-activation gene 3 (LAG3) correlated with partial response (PR) or no response (NR). Patients who achieved complete remission (CR) or partial response with highly active T-cell products (PR_TD_) had a lower expression of exhaustion markers (17). Similarly, the abundance of exhausted-like CD8+ T-cell clones correlated with clinical response failure to bispecific T-cell engagers in patients with MM (18). Furthermore, RRMM patient profile parameters correlated with clinical outcomes in idecabtagene vicleucel (ide-cel) CAR-T therapy studies: higher frequency of T-cells in isolated peripheral blood mononuclear cells (PBMCs), increased T-cell proliferation during manufacturing, higher drug product T-cell transduction and potency, higher absolute lymphocyte counts and a longer washout period after alkylator treatment, were all associated with favorable patient responses (7).

Similar to the importance of competent T-cells for T-cell directed therapies in MM, in patients with acute myeloid leukemia who received allogeneic hematopoietic stem cell transplantation (Allo-SCT), detection of severely exhausted bone marrow memory T cells predicted disease relapse (19). Additionally, increased expression of co-inhibitory receptors in leukapheresis and infusion products have been associated with treatment failure (20). Lastly, T-cell exhaustion may be a potential driver of atypical and otherwise rare infections such as *Pneumocystis jirovecii* pneumonia, cytomegalovirus, or *Aspergillus* species, and can be associated with significant mortality (21, 22). Collectively, these results support the hypothesis that the success of CAR-T and bispecific antibody therapy, could depend on intrinsic T-cell competence and fitness.

## Negative impactors of T-cell health

**Figure 1:**
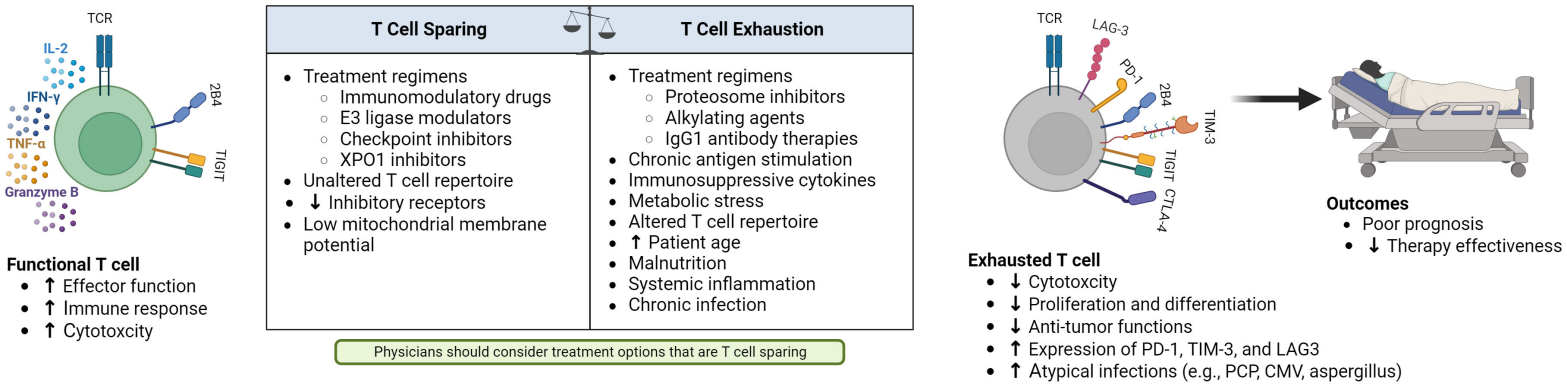
Factors contributing to changes in functional and exhausted T cells, and the resulting poor outcomes of patients with hematological malignancies and T-cell exhaustion.

T-cell fitness can be negatively affected by multiple factors, including prior treatments, increased age, malnutrition, and systemic inflammation (**Figure 1**). Alkylating agents such as cyclophosphamide, commonly used in MM management, impair proliferation and blunt functional activity of T cells (8). The alkylator bendamustine, used to treat hematological malignancies and solid tumors, is associated with decreased naïve T cell counts and impaired T-cell directed cytotoxicity (23, 24). Bendamustine use before immunotherapy negatively affects the clinical outcomes of CAR-T therapy, causing prolonged lymphopenia that can result in serious and fatal infections (25). The lymphocytic populations of these patients, therefore, require careful clinical monitoring (26). Additionally, alkylators used before CAR-T therapy in the pivotal phase 2 KarMMa trial, which investigated B-cell maturation antigen (BCMA)-directed CAR-T therapy, ide-cel, in triple-class exposed patients with RRMM, showed a detrimental effect on the apheresis of PBMC material up to 6-9 months after the last dose (8). In bispecific antibody therapies, given that most of these agents have been developed with continuous therapy schedules, accumulating data also point to the relevance of treatment-free intervals in functional and transcriptional rejuvenation of T cells (27).

Other therapies may also reduce T-cell fitness. In a retrospective analysis, PI administration resulted in deleterious effects on ide-cel production (7). PIs are known to decrease T-cell health and resistance to viral infections, underpinning the recommendation on herpes zoster prophylaxis prescribing instructions in PI-treated patients (28). IgG1 antibody therapies, such as elotuzumab and daratumumab, kill myeloma cells via antibody-dependent cellular cytotoxicity and are associated with T-cell exhaustion and dysfunction when patients become resistant to these agents (29, 30).

Patient demographic characteristics can also negatively impact T-cell fitness. Advanced age is associated with reduced hematopoiesis, a skew towards myeloid cell production, increased systemic inflammation, and dysfunctional T-cell populations (31). Malnutrition, a systemic condition associated with tumor burden, duration of disease, and impact of prior therapies, affects the immune system, including T cells (32). In patients with a poor nutritional state, the thymus atrophies, increasing circulating, immature T cells and T-cell numbers decrease with diminishing intrathymic cell proliferation (33).

## T-cell fitness and mitochondrial metabolism

As key regulators of T-cell metabolism, mitochondria affect T-cell activation, function, and survival, and are important in MM disease progression and drug resistance (34, 35). Whereas low mitochondrial membrane potential (Δψm) is associated with reduced expression of exhaustion markers, lower levels of reactive oxygen species (ROS), and decreased deoxyribonucleic acid damage, high T-cell Δψm is associated with increased production of ROS, reducing T-cell mediated anti-tumor immunity (35). For example, patients with CLL and ALL treated with CAR-T have different therapeutic outcomes (35). Patients with relapsed and refractory ALL had a high remission rate whereas patients with CLL had a lower clinical response rate. This was partly attributed to metabolic impairment of T cells in CLL where resting CD8+ T cells showed lower glucose transporter 1 (GLUT1) expression, increased Δψm after stimulation, and increased mitochondrial ROS (35). In patients with CLL who received CAR-T, phenotypes and genotypes associated with T cells in different states of activation were observed across patients with CR, PR, and NR. Acquired T-cell dysfunction could therefore contribute to a limiting response in CAR-T therapy (35).

Other MM treatments such as the alkylator melphalan, administered in high doses before autologous-SCT, perturbs the composition of the T-cell compartment and drives substantial metabolic remodeling with significant increases in Δψm, thereby reducing T-cell fitness (36, 37). Additionally, chronic infections, including those from disease or immunosuppression therapy can shift T-cell metabolism from that observed in acute infections to an exhausted or dysfunctional phenotype that allows for lowered levels of pathogen control. This shift encompasses metabolic reprogramming including reduced mitochondrial and glycolytic metabolism through the regulation of mammalian target of rapamycin (mTOR) expression and transforming growth factor (TGF)-β-mTOR signaling (38, 39). Suppression of mTOR through TGF-β signaling is a critical regulator of metabolism in precursors of exhausted T cells, which self-renew and continuously generate exhausted effector T cells. Early, transient suppression of mTOR in the precursors of exhausted T cells improved long term T-cell responses in chronic infection mouse models (39).

## T-cell engaging therapies to mitigate T-cell exhaustion

Several factors can increase T-cell fitness (**Figure 1**). Known T-cell stimulating agents, IMiDs, increase proliferation cytokine signaling by several hundred-fold (40). While IMiDs are a transformative therapy in MM, negative consequences of T-cell overstimulation have been observed through induction of graft versus host disease when Allo-SCT recipients received lenalidomide as a maintenance therapy (41, 42). IMiDs and cereblon E3 ligase modulators can improve the efficacy of CAR-T *in vitro* and *in vivo* but there are concomitant specific toxicities such as cytopenias and cytokine release syndrome, perhaps due to T-cell overactivation (43). Current trials are testing the combination of IMiDs with CD19 or BCMA CAR-T therapy in diffuse large B-cell lymphoma and MM (43, 44).

In contrast, checkpoint inhibitors were devised to override T-cell dysfunction or exhaustion due to chronic antigen exposure and suppression by tumor cells (45). However, T-cell overstimulation may be a concern and safety concerns halted earlier trials of checkpoint inhibitors combined with IMiDs in MM (46, 47). T-cell engagement also plays a critical role in two new classes of MM agents: bispecific antibodies and CAR-T therapy. CAR-T therapy overcomes immunological tolerance from recognition of self-antigens by combining effector function antigen recognition in a non-MHC restricted manner (48). The Food and Drug Administration (FDA) approved the use of two different autologous BCMA-directed CAR-T products based in part on the remarkable efficacy of these therapies, despite eventual relapse in most patients (49). Bispecific antibodies overcome the requirement of tumor-associated antigen (TAA) recognition via T-cell receptor by linking two antibody fragments that recognize distinct epitopes on the TAAs and the T-cell surface (13). Teclistamab was recently approved by the FDA as the first bispecific antibody for the treatment of adult patients with RRMM (50). Subsequent studies showed that T-cell engaging salvage therapies appear to maintain the pronounced clinical activity of the BCMA-directed CAR-T therapy (51).

## XPO1 inhibitors for T-cell engagement

XPO1 inhibitors are anti-neoplastic drugs that inhibit XPO1, also known as chromosomal region maintenance 1 (CRM1). XPO1 inhibition causes nuclear retention of critical tumor suppressor proteins such as p53, p21, forkhead box protein A2, and others, facilitating their activation (52, 53) and can prevent oncoprotein translation by inhibiting export of certain oncogene mRNAs that interact with eukaryotic translation initiation factor 4E (54, 55). Additionally, XPO1 inhibitors have anti-inflammatory properties, facilitating a favorable immune microenvironment for effector T cells (56). Specifically, XPO1 inhibition activates several anti-inflammatory, antioxidant, and cytoprotective transcription factors, including inhibitor of κB-α (IκB-α), peroxisome proliferator-activated receptors γ (57), and retinoid X receptor α (58). As the nuclear factor κB (NF-κB) inhibitor, IκB-α, is an XPO1 cargo, selinexor treatment traps IκB-α in the nucleus protecting it from proteasome degradation and inhibiting NF-κB p65. In a sepsis mouse model, oral selinexor reduced cytokine storm-associated inflammatory cytokine secretion, the number of peritoneal cavity macrophages, polymorphonuclear neutrophils, and increased survival (59). *In vivo* research with selinexor demonstrated anti-inflammatory and anti-viral activity following SARS-CoV-2 infection (56). Research with another XPO1 inhibitor, verdinexor, against syncytial respiratory virus (60, 61) and H1N1 influenza (swine flu) (62) found similar results, including decreased cytokine production and viral titer analysis. Finally, the XPO1 inhibitors eltanexor and verdinexor had beneficial effects in zebrafish and Duchenne muscular dystrophy mouse models (63).

## Pre-clinical evidence of T-cell impact by XPO1 inhibitors

**Figure 2:**
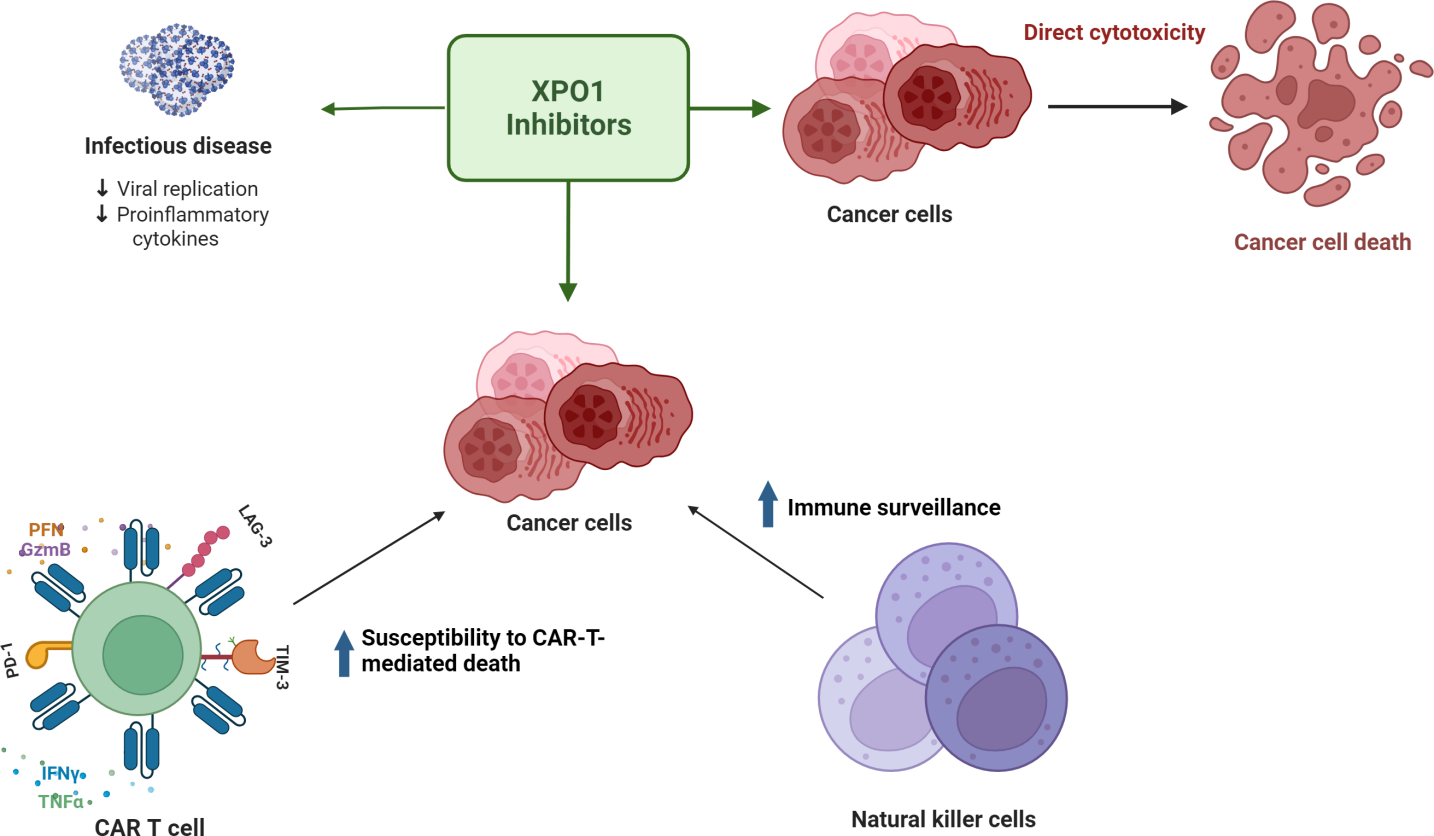
XPO1 inhibitors have direct cytotoxic effects on tumor cells, decrease inflammation in infectious disease, and may facilitate a favorable immune microenvironment for effector T cells to combat T-cell exhaustion.

XPO1 inhibitors have shown potential *in vitro* and *in vivo* in maintenance of T-cell homeostasis and exhaustion mitigation. **Figure 2** summarizes the potential ways XPO1 inhibitors may combat T-cell exhaustion. Combination studies assessing XPO1 inhibitors and CD19-directed CAR-T cells have shown that pretreatment of target cells with an XPO1 inhibitor increases the subsequent cytotoxicity of the CAR-T cells towards those targets and reduces expression of T-cell exhaustion markers including LAG-3, PD-1, and Tim-3 (10, 64). Research using mouse models demonstrated that the frequency of the selinexor dosing schedule was an important factor in the maintenance of T-cell homeostasis (9). A weekly dose of 15 mg/kg preserved nearly normal immune function whereas a thrice-weekly dose of 15 mg/kg or 7.5 mg/kg hindered CD8+ T-cell and B-cell development (9). In a syngeneic immunocompetent B16 mouse model of melanoma, twice weekly dosing with 10 mg/kg selinexor increased the fitness of exogenous tyrosine related protein 1 (TRP1) CD8+ T cells, reduced expression of PD-1, Tim-1, and CD44, and did not impact levels of endogenous tumor-infiltrating lymphocytes (9). Both interferon-γ and granzyme B increased in exogenous and endogenous CD8+ T cells, suggesting improved effector functions (9). Despite transient inhibition of T-cell activation, normal T-cell function was observed when selinexor was administered at clinically-relevant doses and schedule intervals (9). Similarly, a mouse model of non-Hodgkin’s lymphoma showed that biweekly administration of 10 mg/kg selinexor followed by non-XPO1 inhibitor exposed CD19 CAR-T injection significantly reduced the tumor burden at 4-6 weeks compared with CD19 CAR-T injection to non-selinexor treated mice or selinexor alone. This suggests that selinexor sensitized tumor cells to CD19 CAR-T-mediated cytotoxicity; however, the mechanism remains to be elucidated (10).

## Clinical data with XPO1 inhibitors

Recent clinical advances regarding T-cell engagement therapies fall into the pre- and post-anti-BCMA targeting therapy space. Retrospective trial data suggest that tumor cells pretreated with XPO1 inhibitors are more susceptible to T-cell killing. In four clinical studies (STORM, STOMP, BOSTON, XPORT-MM-028), the use of selinexor treatment before anti-BCMA therapy in heavily pretreated MM patients was not associated with inferior response to the anti-BCMA agents (65). The majority of treatment comprised the antibody-drug conjugate (ADC) belantamab-mefadotin and a median overall survival from non-cellular anti-BCMA (NCA) treatment initiation was 12 months (95% CI: 9.4, NE) with a median follow-up of 7.8 months. Median time to discontinuation of NCA treatment was 4.4 months (95% CI: 2.1 NE). In this cohort of heavily pretreated patients with MM who received a selinexor regimen before NCA, overall survival was in the range of 1 year, akin to historical results seen with ADCs. Thus, the 8-week median time between administration of selinexor and NCAs suggested that selinexor combination with various partner agents, did not negatively impact overall survival with subsequent NCA therapy (65). An analysis of salvage treatments from 79 patients following relapse after BCMA CAR-T therapy showed that additional T-cell engaging therapies, including selinexor, may contribute to survival (51). Specifically, of 79 patients, selinexor was given as a first-line or subsequent salvage treatment in 5 and 15 patients respectively. ORR was 40.0% (2 of 5 patients) and 21.4% (3 of 14 patients) and VGPR was also 40.0% (2 of 5 patients) and 21.4% (3 of 14 patients) for selinexor as a first-line and as a subsequent salvage treatment, respectively. First-line salvage treatments had a progression free survival (PFS) of 9 months, including those treated with selinexor (51). In a second study, selinexor paired with low-dose dexamethasone with or without PIs (bortezomib or carfilzomib) resulted in 1 unconfirmed CR, 2 VGPR, 3 PR or 1 minimal response in patients who had exhausted other treatment options for rapidly progressing disease and who had progressed after CAR-T therapy (66). A third study focused on selinexor efficacy in 11 heavily pretreated patients predominantly receiving anti-BCMA-ADC therapy prior to selinexor-containing regimes (11). Compared to previous anti-BCMA therapies, selinexor-containing regimens achieved durable responses with numerically higher ORR (63.6% vs 50.0%), clinical benefit rates (81.8% vs 50.0%), and 6-month PFS (75% vs 12%), despite administration later in the treatment course. Together, these results suggest potential efficacy in selinexor use after failing anti-BCMA therapy. Future prospective clinical studies will explore whether the efficacy is related to the XPO1 inhibitor effect on T-cell health using dynamic T-cell population endpoints.

## Summary and future directions

CAR-T therapy and bispecific antibodies show promise in the treatment of hematologic malignancies, but efficacy is likely dependent on the status of a patient’s immune system, tumor microenvironment, and prior treatment history (4, 15, 67). T-cell exhaustion, driven by age, disease burden, and prior cancer treatment, is a critical limiting factor of these therapies (5). Alkylating agents and PIs have been shown to decrease T-cell fitness (8) whereas XPO1 inhibitors and IMiDs, despite overstimulation risks, may promote T-cell health (9, 64, 68). As such, selinexor-IMiD combinations, which have shown potential in the treatment of RRMM, may be of interest (69–71). The challenge in clinical practice is to maintain CAR-T effector or bispecific antibody therapy function and persistence to achieve clinical potency, while simultaneously balancing the risk of immediate and delayed infections with best prophylactic approaches (4, 72).

Oral small molecules such as XPO1 inhibitors offer the potential to improve T-cell fitness and augment cancer cell immune susceptibility without negatively impacting T-cell health. Towards this end, downstream partners engaged by XPO1 inhibitors may be elucidated through analysis of serologic and marrow immunophenotypic signatures, paired sequencing data from primary myeloma patient samples on active XPO1 inhibitor therapy, and therapies that have the potential to hamper T-cell fitness. Particular emphasis may be placed on expression profiles and activity of inhibitory pathways (e.g., PD-1, LAG3 and/or TIM3), the extent of activation and/or suppression of CD8 T cell effector functions (i.e., IFNγ and granzyme B) as well as other transcriptional signatures of proliferation (4, 9). In addition, factors such as substantial metabolic remodeling with significant increases in mitochondrial membrane potential, which can reduce T cell fitness, may also play a role and should be assessed (5). Further clinical studies that include optimizing dosing schedules to prime the T-cell repertoire or post-T-cell therapy to aid the persistence of CAR-T activity are needed to evaluate the impact of XPO1 inhibitors on CAR-T and MM cell immune susceptibility. Such clinical and real-world data may further elucidate how disease and host factors, including exposure to prior therapies, interplay to inform optimization of long-term disease control.

## Author contributions

MB: Conceptualization, Writing – original draft, Writing – review & editing. AB: Conceptualization, Writing – original draft, Writing – review & editing. CW: Conceptualization, Writing – original draft, Writing – review & editing. TM: Conceptualization, Writing – original draft, Writing – review & editing.
